# Efficient dendritic learning as an alternative to synaptic plasticity hypothesis

**DOI:** 10.1038/s41598-022-10466-8

**Published:** 2022-04-28

**Authors:** Shiri Hodassman, Roni Vardi, Yael Tugendhaft, Amir Goldental, Ido Kanter

**Affiliations:** 1 Department of Physics, Bar-Ilan University, 52900 Ramat-Gan, Israel; 2 Gonda Interdisciplinary Brain Research Center, Bar-Ilan University, 52900 Ramat-Gan, Israel

**Keywords:** Biophysics, Computational biology and bioinformatics

## Abstract

Synaptic plasticity is a long-lasting core hypothesis of brain learning that suggests local adaptation between two connecting neurons and forms the foundation of machine learning. The main complexity of synaptic plasticity is that synapses and dendrites connect neurons in series and existing experiments cannot pinpoint the significant imprinted adaptation location. We showed efficient backpropagation and Hebbian learning on dendritic trees, inspired by experimental-based evidence, for sub-dendritic adaptation and its nonlinear amplification. It has proven to achieve success rates approaching unity for handwritten digits recognition, indicating realization of deep learning even by a single dendrite or neuron. Additionally, dendritic amplification practically generates an exponential number of input crosses, higher-order interactions, with the number of inputs, which enhance success rates. However, direct implementation of a large number of the cross weights and their exhaustive manipulation independently is beyond existing and anticipated computational power. Hence, a new type of nonlinear adaptive dendritic hardware for imitating dendritic learning and estimating the computational capability of the brain must be built.

## Introduction

**Figure 1:**
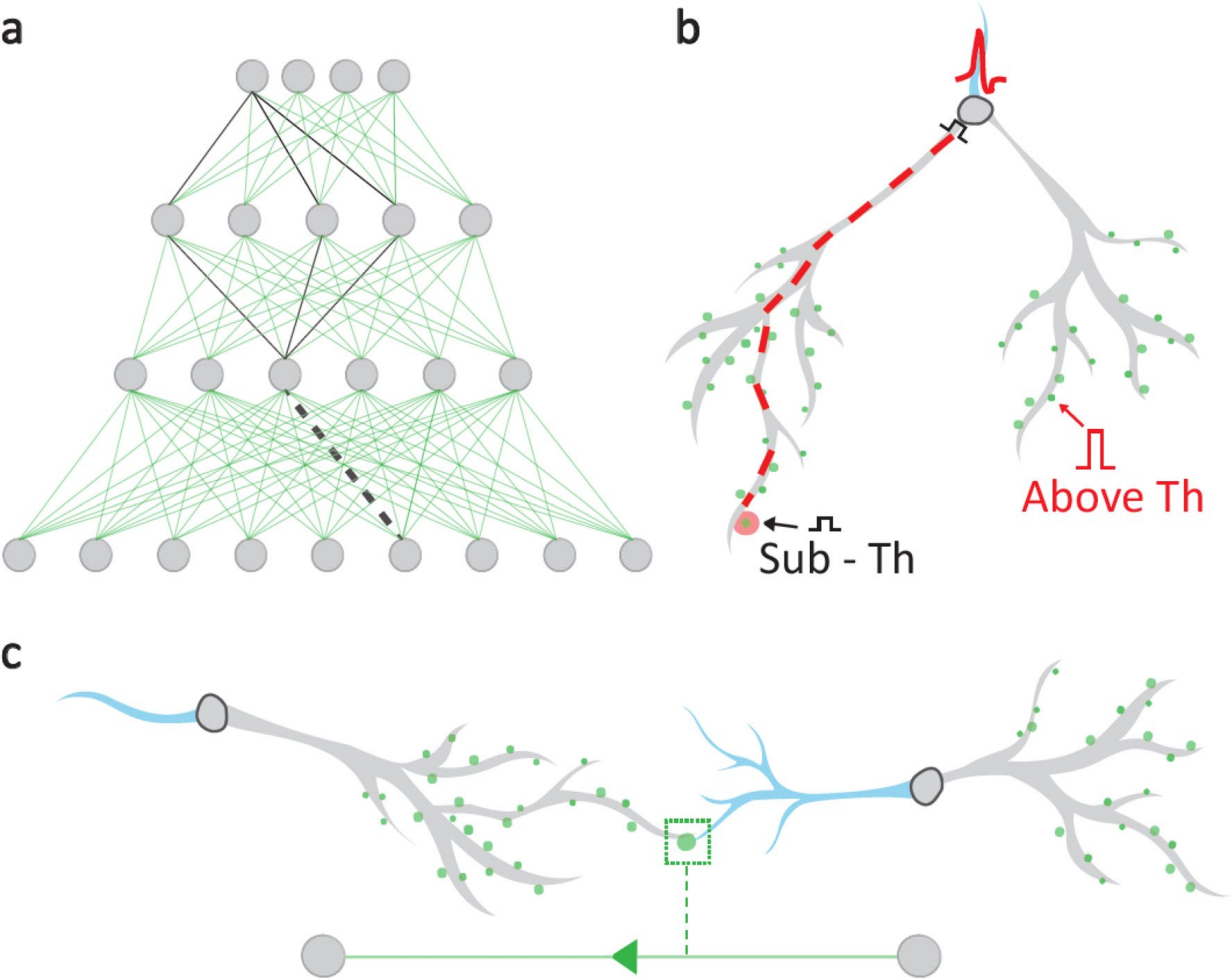
From a biological learning scheme to ANNs. (**a**) Scheme of two hidden layers of a feedforward network, where several routes (black) connect an output unit to a weight (black dashed line). (**b**) Scheme of a neuron (node in **a**) with two dendrites (gray), an axon (blue), and multiple synapses (green circles). A sub-threshold synaptic stimulation (black) via the left dendrite arrives at the soma after a spike (red) was generated through an above-threshold synaptic stimulation via the right dendrite, thereby strengthening the left synapse (enlarged red circle) via a backpropagation signal (red dashed line). (**c**) Biological scheme of the right neuron connecting to the left neuron via axon/synapse/dendrite (blue/green-circle/gray). Artificial scheme as in **a** (bottom).

A popular method for training artificial neural networks is related to synaptic plasticity (SP), which governs the brain adaptation mechanism^1^ and where the connection strength between two neurons is modified following their relative activities^2,3^. This local adaptation is the foundation of the learning process of artificial neural networks (ANNs)^4^. Classification and representation of practical problems require feedforward networks comprising hidden layers to be trained^5^, which mediate between input and output units^6^ (Fig. 1a). This is how deep learning (DL), as a subfield of machine learning, originated, which now outperforms humans in addressing difficult problems^7,8^, such as face recognition, and games (e.g., chess and go)^9–11^.

In a supervised learning scenario, a feedforward step is initially performed. An input is presented to the feedforward network, and the distance between the current and desired outputs is computed using a given error function. The backpropagation (BP) procedure is utilized in the next step, where weights are updated to locally minimize the error function^5,12^. This procedure is repeated several times over the training set, until a desired test error is achieved.

Traditionally, this DL technique stems from the dynamics of the human brain, however, these two scenarios are intrinsically different^13^. The main reason for this assumption is that BP procedure is biologically implausible, as it changes the weight in a non-local manner. The number of routes between an output unit and a weight, via hidden layers, is typically large (Fig. 1a). Each route contributes to a weight modification following a combination of all weights, and nonlinear nodal activation functions along the route. The enormous transportation of precise weight information can be performed effectively using fast and parallel GPUs. However, they are evidently beyond biological realization.

## Results

### Long-lasting assumption of SP

The current version of imprinted SP is based on mutual, time-dependent activity by pre- and post- synaptic neurons^3^ (Fig. 1b). This is supported by experimental evidence showing adaptation typically consumes tens of minutes and incorporates considerable fluctuations^14^. Its main mechanism is the BP signal along the dendrite, which serves as a byproduct of the spiking neuron to its axon ^14–16^ (Fig. 1b). The long transportation distance from the soma to the synapse, along varying conducting dendritic brunches, is expected to be noisy and to fluctuate. The main complexity of the SP assumption is that synapses and dendrites are connected in series (Fig. 1c). However, existing experiments that stimulate two presynaptic neurons, or pre- and post-synaptic neurons, cannot pinpoint the significant imprinted adaptation location, whether it is located at the synapse or at the dendrite. Although there is a consensus on temporal adaptation in synaptic boutons and spines, following their recent stimulation patterns, their time independent imprinted adaptation without further stimulations is in question. In addition, assuming imprinted SP, one cannot exclude from current experiments fast and significant enhanced adaptation in the dendrites connected in series to the synapses.

### Dendritic learning

Results of recent experiments indicate that fast and enhanced adaptation occurs when two dendrites are mutually trained, similar to the slow adaptation currently attributed to the synapses^17^. This phenomenon differs from dendritic computation^18^ based on static dendritic features. Its timescale depends on the training frequency and can be reduced to several seconds only^19^. Although the results pose a question on SP, current experiment results cannot exclude slow and noisy SP in parallel to fast dendritic adaptation. Experiments also indicate that certain dendrites demonstrate forward and backward action potentials and nonlinear dendritic excitability, which resembles spike waveforms^20–22^. We begin with the simulation results, where experimental results supporting adaptation within a dendrite are briefly presented.

### Realization of DL by a single neuron

**Figure 2:**
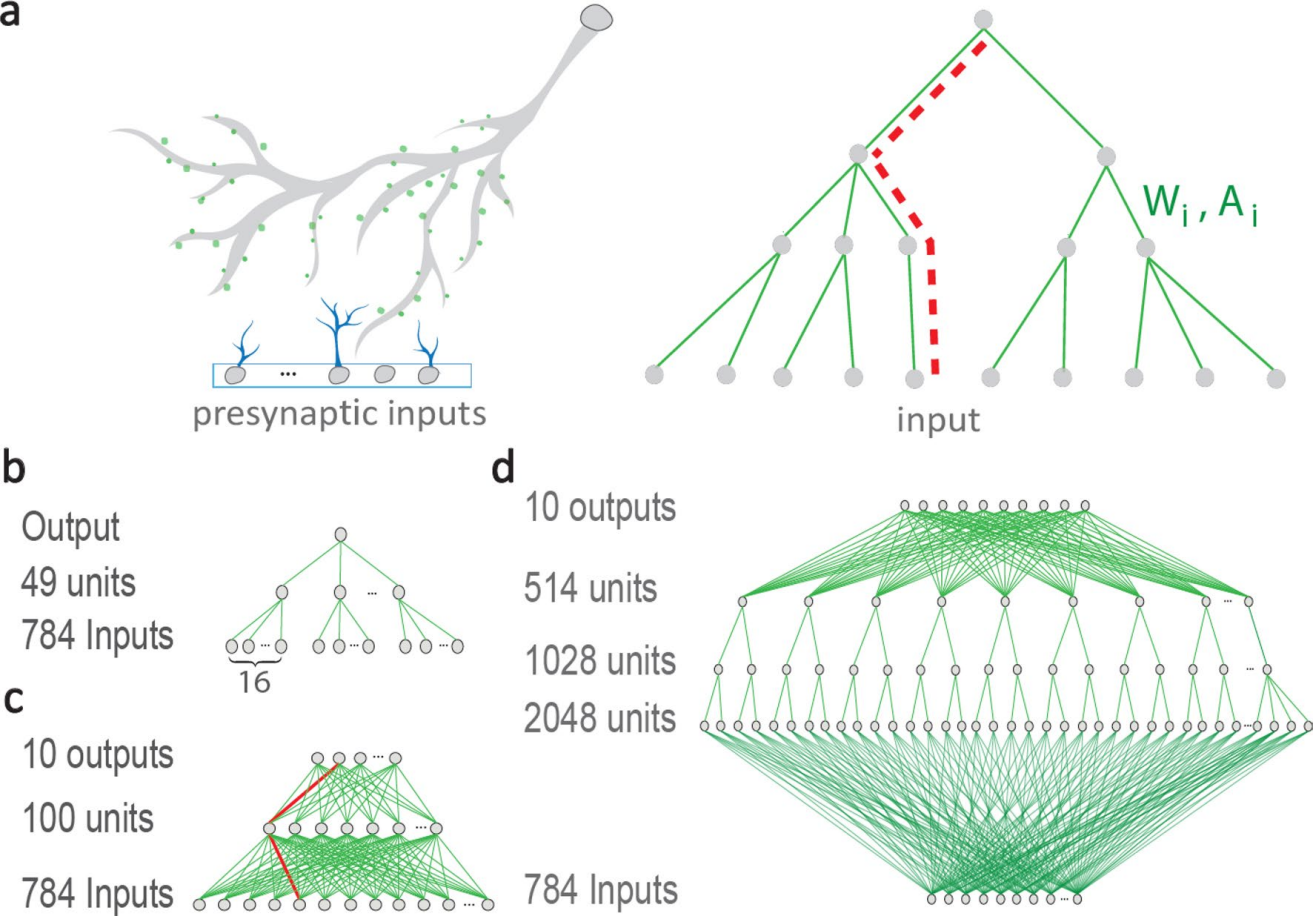
From dendritic learning to BP on FFTNs. (**a**) Neuronal scheme of a dendritic tree (left), synapses (green circles), and their presynaptic input neurons (bottom gray circles) and axons (blue). Similar tree scheme (right) with a single route (red dashed line) from the output to each input. Each tree segment is characterized by weight W_i_ and nonlinear amplification A_i_. (**b**) Trained FFTN to recognize one handwritten digit. (**c**) Fully connected FFTN with 10 output units, one for each handwritten digit. (**d**) An example of FFTN with respect to weights, where each weight is connected to an output unit via one route only.

We recently experimentally examined dendritic adaptation by mutually training two dendrites^17^. However, the adaptation sites along the dendrites were obscure. Here, we assumed an adaptive strength for each dendritic segment, where each segment additionally functioned as a nonlinear amplifier^23–31^ (Fig. 2a, right).

Implementing BP on a tree architecture was simpler as each weight was influenced by an output unit via one route only (Fig. 2a). A weight change was accumulated backward from the output unit along the route, where temporarily only a nodal state and its successive weight were required, no long-term memory was needed. The quantitative results of such a tree BP (TBP) on a feedforward tree network (FFTN) were presented for the recognition of 10 handwritten digits derived from the Modified National Institute of Standards and Technology (MNIST) database^32^.

The architecture we first examined comprised 10 FFTN identifiers, where each network consisted of 784 (28 × 28) input units, 49 hidden units connected to 16 non-overlapping input units each, and one output unit (Fig. 2b). Each FFTN was trained independently to identify one digit. For the selected digit, the output is trained toward 1; otherwise, toward 0. The predicted test digit was selected as the FFTN with the maximal output value. The training parameter optimization (described in "Methods") resulted in a ~ 0.047 test error. Generalization of each FFTN to several FFTNs trained independently using different initial conditions and with a soft committee output (described in "Methods") resulted in a ~ 0.034 test error. We note, that training only the weights from the input to the hidden units results in an optimized test error greater than 0.47 for fixed, uniform or random, weights to the output unit. This significant increase in the test errors indicates the importance of the training of the entire FFTN (Fig. 2b), besides weights from the inputs functioning similar to SP. This result is much below the success rates of a linear classifier^33^ and is attributed to the nonoverlapping receptive fields of the nonlinear hidden units, where each one is influenced by a small subset of the inputs, and for the nonlinear activation functions of the hidden units with fixed output weights.

Training a fully connected architecture network, with 100 hidden units and 10 output units (Fig. 2c) results in a test error of 0.018 only^34^ (see "Methods"). Each output unit in this architecture is connected to an input unit via multiple routes, thus violating the tree structure in terms of nodes. Nevertheless, each output unit was connected to a weight via one route only (Fig. 2c), and thus the principle of TBP holds. In general, TBP holds for fully connected input/output layers to their nearby layers and tree structure elsewhere (Fig. 2d).

The biological realization of TBP on these architectures (Fig. 2c) poses the following two conditions: First, each weight that connects the input and hidden nodes must be updated 10 times, according to the current output values of the 10 output nodes. These updates can be realized asynchronously using, for example, different delays for each output unit. Second, error function $\epsilon$ is a summation of individual errors of each output unit

$$\epsilon = \mathop \sum \limits_{i = 1}^{10} \epsilon_{i} = \mathop \sum \limits_{i = 1}^{10} \left[ {O_{i} - O_{i}^{desired} } \right]^{2}$$

as exemplified for the quadratic error function, where $O_{i} /O_{i}^{desired}$ denotes the output and desired output, respectively. This property holds also for the cross-entropy cost function used in this study (see "Methods"). Note that an output node in Fig. 2c, for instance, can biologically imitate a neuron with a single dendritic tree, using an additional output node connected in series to the current one via a single weight. This additional weight represents the dendritic route. In principle, the entire network (Fig. 2c) might also be represented by a neuron with a ramified dendrite, however, it requires a more complex structure to the output layer and will be discussed elsewhere.

### Realization of input crosses by a single neuron

**Figure 3:**
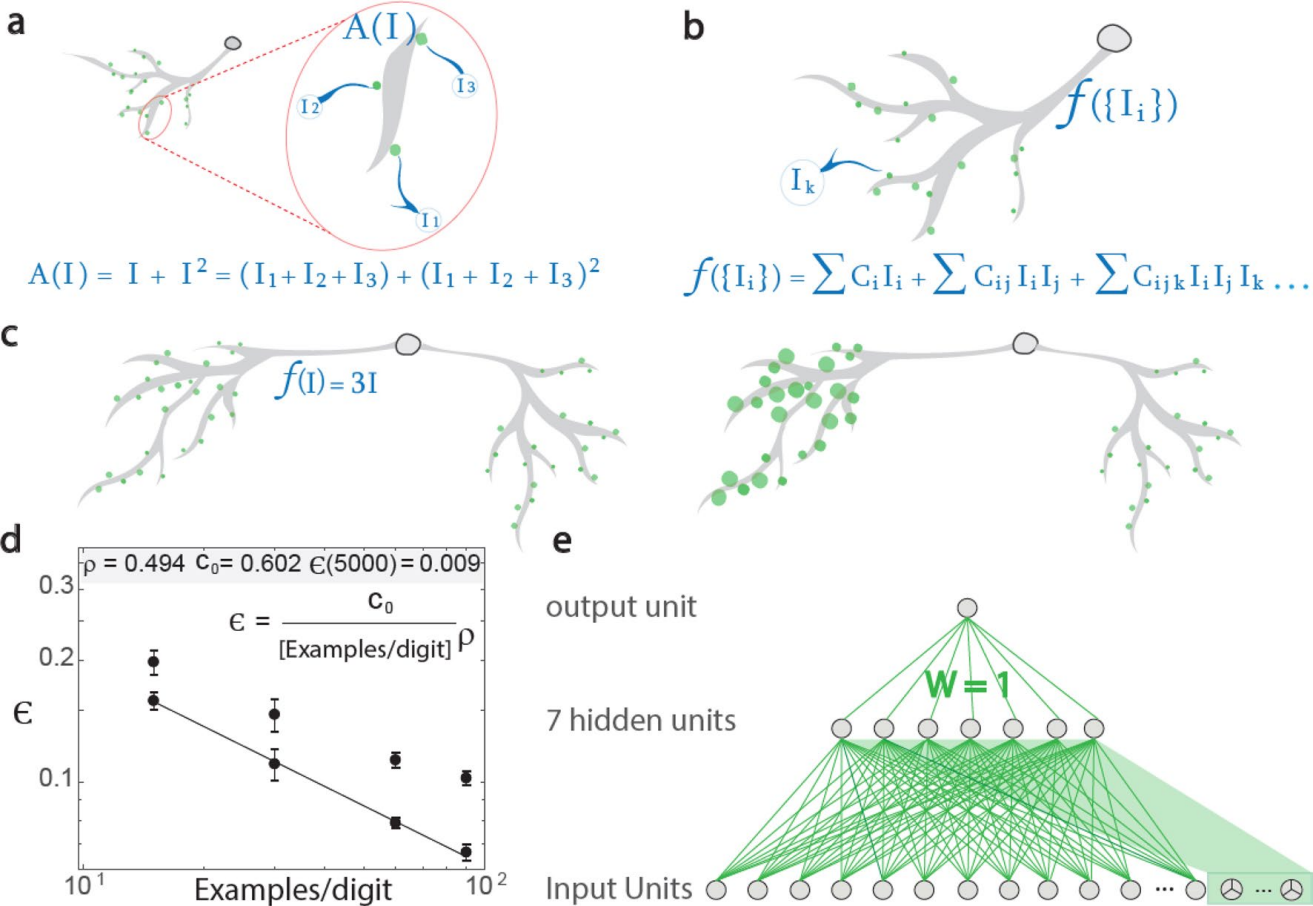
Biological mechanism for self-emergence of input crosses. (**a**) Zoom-in on three inputs (i.e., $$I_{1} , I_{2} , I_{3} )$$ dendritic segment, with nonlinear amplification $$A\left( I \right) = I + I^{2}$$, resulting in input crosses. (**b**) Combining all synaptic influx inputs $$I\left( {\left\{ {I_{k} } \right\}} \right)$$ results in higher-order input crosses. (**c**) Equivalence between one amplified dendrite, $$f\left( I \right) = 3I$$ (left) and where amplification is shifted to its synapses (larger green-circles, right). (**d**) Log–log scale of optimized test errors for the architecture presented in Fig. 2c with additional 10,000 input crosses trained over 15, 30, 60 and 90 examples/digit and a power-law fit (line), and without input crosses (circles). Standard deviation obtained from 10 samples with different initial conditions, i.e. weights and examples. (**e**) Hebbian learning identifier for a digit, consisting of a committee of seven perceptrons, each with 10,000 input crosses of order 3 (green background).

Input crosses that represent higher-order correlations among input nodes can enhance success rates^34–37^. However, their biological realization is questionable as no morphological evidence to multiple connections of several axons to one synapse exists, which might accomplish input crosses^38^. In addition, multiple connections of one axon to a neuron is infrequent^38^ and cannot accomplish input crosses. Nevertheless, the byproduct of multiple inputs to a nonlinear dendritic segment amplifier accomplishes input crosses (Fig. 3a). Their order and number are further enhanced by nonlinear dendritic segment amplifiers closer to the soma, which typically incorporate a vanishing number of newer inputs (Fig. 3b). This amplification represents a non-local adaptation mechanism, as dendritic segment amplification is equivalent to simultaneous amplification of influx through all its incoming synapses (Fig. 3c).

The addition of 10,000 input crosses among three inputs for each hidden unit, in a micro-canonical manner^34^ (described in "Methods"), enhances success rates by ~ 3% for small training datasets comprising 15, 30 and 60 examples per digit (Fig. 3d). Maximization of success rates using multiple input crosses at the maximal training dataset (i.e., 50,000 examples) is a heavy computational task. Nevertheless, a power-law extrapolation of the obtained success rates from small training datasets

$$\epsilon = \frac{{c_{0} }}{{\left[ {\frac{examples}{{digit}}} \right]^{\rho } }}$$

to the maximal dataset results in a  ~ 0.01 error rate (Fig. 3d), which outperforms the extrapolation of the error rate without input crosses, ~ 0.018,^34^ which is now directly confirmed (see "Methods").

Using input crosses among three inputs achieved slightly better success rates and faster convergence times than using input crosses between two inputs^34^. This observation might indicate the importance of higher-order input crosses, where their improvement might stem from the phenomenon of strong first-order phase transition for systems with higher-order multi-spin interactions^39^.

### Hebbian learning using a single neuron

The TBP procedure simplifies the biological realization of DL. However, the necessity to precisely calculate derivatives of activation functions and their products with nearby weights is beyond known biological hardware capabilities. Thus, using tiny imprecise updates that result in accumulated small additive and multiplicative noise to the TBP procedure is expected to only slightly decrease the success rates. However, their enhancement entails unavoidable significant deterioration in obtained success rates. Here, we have presented another possible solution based on the perceptron local learning algorithm^40^.

The architecture consisted of 10 FFTNs, each of which consisted of seven perceptrons, with an additional 10,000 input crosses (see "Methods"). The output of each FFTN was a committee of the seven perceptrons, connected to the output with unit weights (Fig. 3e). Each FFTN was trained independently using the least action algorithm^41,42^ to recognize one digit. For the selected digit, the output is trained to be 1; otherwise, -1. The perceptron learning step was performed only when the number of perceptrons with the correct output was less than 5. The step was realized on the perceptron with a wrong output and a minimal absolute local field. Thus, a test error of ~ 0.029 was obtained (described in "Methods"). Note that the least action algorithm requires the knowledge of output local fields of all perceptrons, a non-local decision, but their number is small.

### Experimental results supporting intra dendritic adaptation

**Figure 4:**
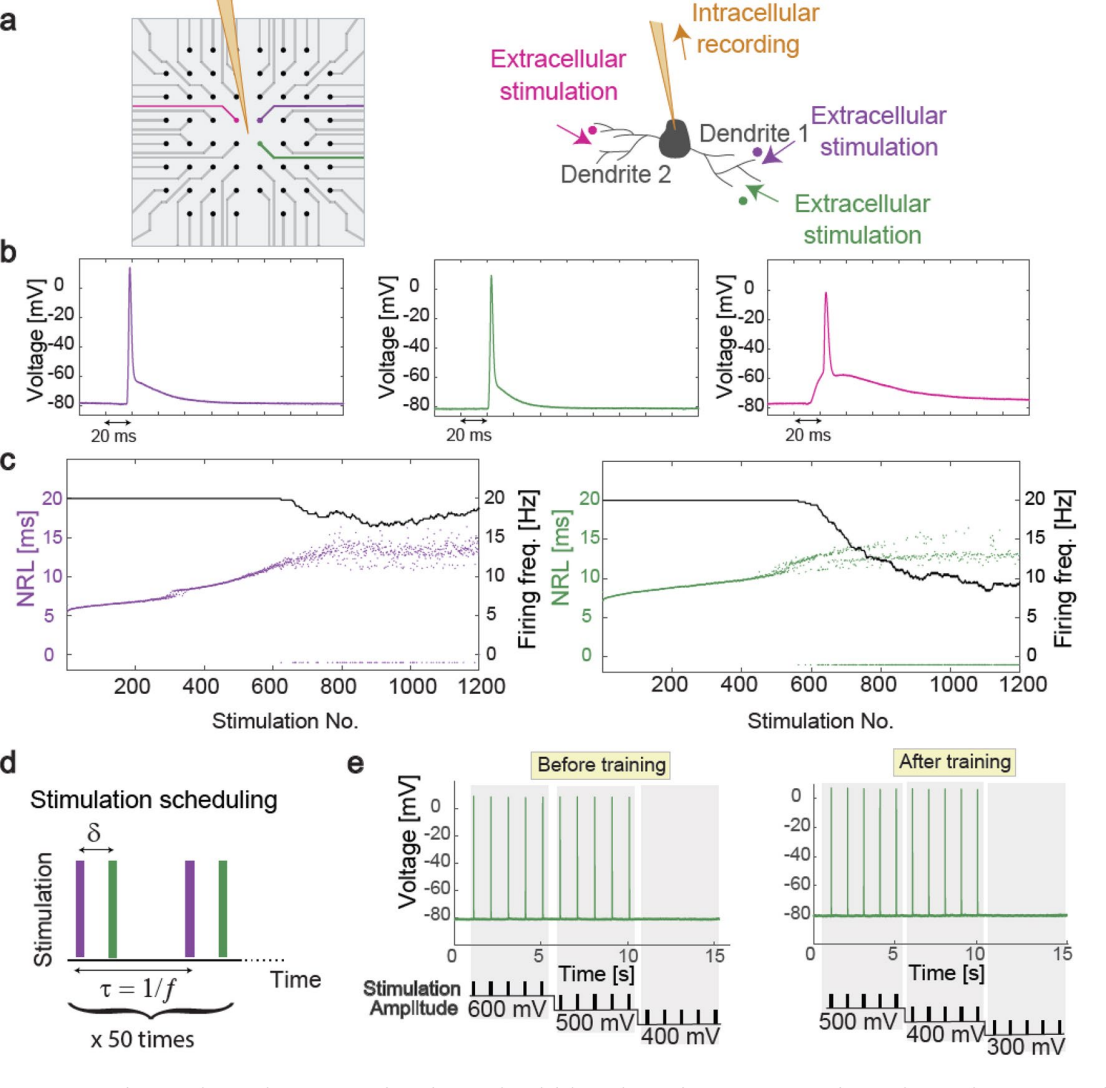
Experimental results supporting intra dendritic adaptation. (**a**) 60 micro-electrode array and scheme of an intracellular electrode (orange) and three nearby extracellular electrodes (i.e., pink, purple, and green) (left). Scheme of a patched neuron recorded intracellularly (orange), two stimulating extracellular electrodes (i.e., green and purple) adjacent to one dendrite, and third electrode (pink) near a different dendrite (right). (**b**) Two stimulating extracellular electrodes (i.e., green and purple) generate similar intracellularly recorded spike waveforms, which differ from the third one (pink). (**c**) Neuronal response latency, measuring the time lag between an extracellular stimulation and its corresponding evoked spike, for the green and purple extracellular electrodes, stimulated at 20 Hz. Response failures are denoted at − 1. The effective firing frequency is presented using sliding windows of 100 stimulations (black). (**d**) Training scheduling consists of 50 repeated pairs using δ = 4 ms and *f* = 5 Hz. (**e**) Intracellular recordings of threshold estimation by extracellularly stimulating five times at 1 Hz for each stimulation amplitude (bottom) using the green electrode, before and after training.

Recently, new types of experiments have been performed^43^, wherein the synaptic connectivity of neuronal cultures is excluded (see "Methods") and a patched neuron is extracellularly stimulated from several sites using a multi-electrode array (Fig. 4a). The experimental results indicate that a neuron functions as a collection of independent threshold units, with a specific spike waveform for each one^43^. Specifically, the neuron is anisotropically activated following the origin of the arriving signals to the soma, via its dendritic trees^43–45^, and the neuronal spike waveform varies as a function of the stimulation location (Fig. 4b).

These anisotropic properties can demonstrate fast dendritic adaptation^17^, similar to the slow adaptation mechanism currently attributed to synapses^3,46,47^. We used an online method to identify a pair of differing extra- and intra-cellular recorded spike waveforms that represent neuronal activation from two dendritic trees. The training procedure involves pairs of an extracellular stimulation that did not evoke a spike and arrived with a predefined delay, typically a few milliseconds, after (or before) an above-threshold intracellular stimulation. For training at a low frequency (e.g., 1 Hz), a significant effect of adaptation was observed after several minutes and was found to be irreversible for a timescale of tens of minutes^17^. Further, an increase in the training frequency (5 Hz) accelerated neuronal adaptation processes to several seconds only^19^.

The resolution of our experimental setup does not allow to pinpoint the sub-dendritic adaptation sites. Nevertheless, in this work, we presented a support for a dendritic adaptation while two of its branches were trained. We used an online method^43^ to identify a pair of two extracellular electrodes with similar intracellularly recorded spike waveforms, but varying neuronal response latency and different critical firing frequencies^45^ (Fig. 4c), hence represented neuronal activation via different branches of the same dendrite (Fig. 4a). Finally, the neuron was trained using pairs of extracellular stimulations (Fig. 4d), where the stimulation amplitude threshold of one of the electrodes had changed after training (Fig. 4e).

Results indicate an adaption process while stimulating different extracellular electrodes that represent training different routes of the same dendrite (Fig. 4e) and suggest that the adaptation occurs in a sub-route of the trained dendritic tree. This adaptation process is also supported by preliminary results (not shown) where the threshold of the route which is associated with the first stimulation in a pair (purple electrode in Fig. 4d), remains unchanged after the training process. This phenomenon is the inspiration for the TBP scheme presented in Fig. 2. We note that further investigation of the number of sub-dendritic adaptation sites, amplitudes and timescales demands longer measurements with higher resolution experimental techniques.

## Discussion

SP is the core hypothesis of brain learning, and its reality is challenged by the following two aspects: SP as a standalone learning mechanism and in comparison to dendritic learning.

As a standalone mechanism, imprinted SP is a slow and noisy adaptation process, which typically lasts tens of minutes and occurs far from the computational element, namely, the spiking soma. The realization of efficient learning in ANNs using the biological recipe of SP is obscure. In addition, time lags among influx stimulations of the soma via different synapses are a critical parameter that controls the adaptation process. However, these time lags are a function of neuronal response latencies that fluctuate and vary dynamically, following previous activities of connecting neuronal chains^48^. Moreover, synaptic strengths are typically considerably below threshold^49,50^ and many coordinated input timings are required to repeatedly reproduce the same desired neuronal outputs.

A pair of neurons are connected using several elements in a series and in particular synapses and dendrites. The long-established hypothesis states the adaptation occurs in synapses, which is generally supported experimentally by training of pre- and post-synaptic neurons. However, this evidence cannot pinpoint the significant imprinted adaptation sites, without tracing the signal along its inter-neuronal route. Our experiments demonstrated significant dendritic adaptation that emerged at least one order of magnitude faster than the common scenarios for imprinted SP. Currently, one cannot exclude slow, moderate, and noisy SP in parallel to the measured dendritic adaptation. Moreover, the number of dendritic branches is in the order of tens^51,52^, whereas several thousands of synapses exist per dendrite. Hence, dendritic segment adaptation is equivalent to simultaneous adaptation of all its incoming synapses (Fig. 3c). This non-local adaptive process is expected to enhance the signal-to-noise ratio in comparison to SP.

Learning on dendritic trees, where each weight is connected to an output unit via one route only, represents a step toward a plausible biological realization. Tree architectures, although comprising much lesser number of weights, have been demonstrated for the MNIST database to achieve success rates closer to unity, which were previously obtained using more structured DL architectures. This represents the effectiveness of DL when the number of adaptive parameters is in the order of the number of nodes. The realization of dendritic learning using 10 independent FFTN identifiers, one for each digit, and especially using the Hebbian learning rule, might also lead to a better understanding of the biological credit assignment mechanism^13,53,54^.

The emergence of many input crosses as a byproduct of nonlinear amplification of dendritic segments differentiates between the computational power of a single dendrite or neuron from existing CPUs and GPUs. Each dendrite has thousands of presynaptic inputs that generate an exponential number of input crosses as segment signals are propagating toward the soma. For a thousand dendritic inputs, for example, there are O(10^6^) input crosses of order 2 and O(10^21^) input crosses of order 7. Assigning independent weights to such a large number of input crosses and manipulating their strengths via the BP procedure is beyond existing and anticipated computational power. Evidently, this large number of cross weights are not independent. Knowledge of dendritic inputs, dendritic local weights, and nonlinear amplifiers determines all current cross weights and nodal responses. This valid mathematical statement calls for modelling dendritic nonlinear amplification, rather than estimating weights independently through BP. In addition, it calls for the learning of the type of the nonlinear nodal activation functions as an additional tunable parameters, which can control the ratios between induced cross weights and their order. We note that the byproduct of a deep architecture scheme, consisting of more hidden layers and nonlinear activation functions, is the emergence of cross weights, however, the manipulation of each one of them independently is difficult. Thus, the current cost function for training neural networks can be improved by training independently many cross weights, where in addition features of the adaptive nonlinear amplifiers are changed following the learning process. Furthermore, nonlinear sub-segment dendritic amplifications result in non-intuitive phenomena such that amplification is sensitive to the order of dendritic segment inputs. In addition, adaptation of one sub-segment due to a nearby input, might have a decaying effect on the strength of subsequent sub-sequences, which in turn might create nontrivial dependencies between multiple tunable parameters beyond current simulation models. Existing computer hardware that differs from exemplified brain dynamics is distant from the possibility of imitating their learning process and estimating their computational capabilities.

Finally, the experimental support of dendritic adaptation must be refined, using a tradeoff between higher spatial resolution of dendritic segment measurements and long periods of multiple stimulation scheduling. This type of experiment is expected to verify the possible coexistence of SP alongside dendritic adaptation. Qualitative modeling of the main features of dendritic adaptation and their differentiation among various neurons and dendrites are required for understanding of neural network dynamics and their computational capabilities.

## Methods

### Architecture and initial weights, Fig. 2c

The feedforward neural network consisted of 784 input units, 2 hidden layers consisting of 100 units each and 10 output units. Weights between successive layers were fully connected. Each unit in the hidden and the output layers had an additional input from a bias unit^34^. We denote by W^1^ and W^2^ the weights from the input layer to the hidden layer and from the hidden layer to the output layer, respectively. The initial conditions of all weights were randomly chosen from a Gaussian distribution with a zero average and standard deviation (Std) equals 1. All weights were normalized at the initial condition only^19^ such that all input weights to each hidden unit had a zero average and Std equals 1. In addition, the initial value of the bias of each weight was set to 1.

### Input

Each example, $\tilde{X}_{m} , m = 1, 2, \ldots , M$, of the train dataset consisted of 784 pixels, $\tilde{X}_{m,p}$, which their values were in the range [0, 255]. The input, X, of the example $\tilde{X}$, consisted of the original 784 pixels where the average pixel value in $\tilde{X}$ was subtracted from each pixel and the Std was set to 1:

$${\text{X}}_{{\text{m}}} = \tilde{X}_{m} - \frac{1}{784}\mathop \sum \limits_{{{\text{p}} = 1}}^{784} \tilde{X}_{m,p}$$

$${\text{X}}_{{\text{m}}} = {\text{X}}_{{\text{m}}} /std\left( {\tilde{X}_{m} } \right)$$

Furthermore, an input pixel which had an identical value among all the training examples, e.g., had zero variance in all train dataset examples, was set to zero.

### Architecture and initial weights, Fig. 3d

The architecture of this network was similar to the architecture in Fig. 2c, with only one hidden layer and additional 10,000 input-crosses for each hidden unit.

An addition of 10,000 input-crosses was added to the input, ${\text{X}}_{{{\text{k}},{\text{l}},{\text{j}}}}$:

$${\text{X}}_{{{\text{k}},{\text{l}},{\text{j}}}} { } = {\text{X}}_{{\text{k}}} \cdot {\text{X}}_{{\text{l}}} \cdot {\text{X}}_{{\text{j}}}$$

where k, j and l are random indices in the range [1, 784] with corresponding different pixels $X_{k}$ ,${\text{ X}}_{{\text{j}}}$ and $X_{l}$ for a given example. Zero input-crosses in all the train dataset were excluded. Each input-cross was not connected more than once to each hidden unit.

After the above-mentioned initial normalization of all weights, the weights of the input-crosses were rescaled:

$$W_{input \,crosses} = \sqrt {\frac{\# regular \, input}{{\# input \, crosses}}} \cdot W_{input \,crosses} = \sqrt {\frac{784}{{10000}}} \cdot W_{input \,crosses}$$

### Forward propagation

The output of a unit, j, in the first hidden layer for the mth example, for instance, $a_{j,m}^{1}$, was calculated as:

$${\text{z}}_{{{\text{j}},{\text{m}}}}^{1} = \mathop \sum \limits_{{\text{j}}} \left( {{\text{W}}_{{{\text{ij}}}}^{1} \cdot {\text{X}}_{{\text{i}}} } \right) + {\text{b}}_{{\text{j}}}^{1}$$

$${\text{z}}_{{{\text{j}},{\text{m}}}}^{1} = {\text{z}}_{{{\text{j}},{\text{m}}}}^{1} - Amp_{1} \cdot \frac{1}{{{\text{m}} - 1}}\mathop \sum \limits_{{{\text{t}} = 1}}^{{{\text{m}} - 1}} {\text{z}}_{{{\text{j}},{\text{t}}}}^{1}$$

$$a_{{{\text{j}},{\text{m}}}}^{1} = \frac{1}{{1 + {\text{e}}^{{ - {\text{z}}_{{{\text{j}},{\text{m}}}}^{1} }} }}$$

where ${\text{W}}_{{{\text{ij}}}}^{1}$ is the weight from the ith input unit to the jth hidden unit, ${\text{X}}_{{\text{i}}}$ is the ith input, and ${\text{b}}_{{\text{j}}}^{1}$ is the bias induced on the jth unit in the first hidden layer. ${\text{z}}_{{{\text{j}},{\text{m}}}}^{1}$ represents the field propagating from the input layer. Each time we calculated the field, ${\text{z}}_{{{\text{j}},{\text{m}}}}^{1} ,$ we subtracted the accumulative average field for the input layer of the previous $m - 1$ examples, where $Amp_{1}$ is a constant representing the amplitude of reduction. Note that ${\text{z}}_{{{\text{j}},{\text{m}}}}^{1}$ was not modified for m = 1.

The output of the jth unit in the output layer, ${\text{a}}_{{\text{j}}}^{2}$, was calculated as following:

$${\text{z}}_{{{\text{j}},m}}^{2} = \mathop \sum \limits_{{\text{j}}} \left( {{\text{W}}_{{{\text{ij}}}}^{2} \cdot a_{{{\text{j}},{\text{m}}}}^{1} } \right) + {\text{b}}_{{\text{j}}}^{2}$$

$$a_{{{\text{j}},{\text{m}}}}^{2} = \frac{1}{{1 + {\text{e}}^{{ - {\text{Z}}_{{{\text{j}},{\text{m}}}}^{2} }} }}$$

where ${\text{W}}_{{{\text{ij}}}}^{2}$ is the weight from the ith unit in the hidden layer to the jth output unit, and ${\text{b}}_{{\text{j}}}^{2}$ is the bias induced on the jth output unit.

### Back propagation

We used the cross entropy cost function:

$${\text{C}} = - \frac{1}{{\text{M}}}\mathop \sum \limits_{{{\text{m}} = 1}}^{{\text{M}}} \left[ {y_{m} \cdot \log \left( {a_{m} } \right) + \left( {1 - y_{m} } \right) \cdot \log \left( {1 - a_{m} } \right)} \right] + \frac{\alpha }{2\eta }\mathop \sum \limits_{i} W_{i }^{2}$$

where $y_{m}$ stands for the desired labels, $a_{m}$ stands for the current 10 output units of the output layer, and $\eta$ and $\alpha$ are constants. The summation was over all M training examples. The second summation was over all weights of the network.

The backpropagation using the momentum method computes the gradient for each weight with respect to the cost function. The weights and biases were updated according to:

$${\text{V}}^{{{\text{t}} + 1}} = {\upmu } \cdot {\text{V}}^{{\text{t}}} - {\upeta } \cdot \nabla_{{{\text{W}}^{{\text{t}}} }} {\text{C}}$$

$${\text{W}}^{{{\text{t}} + 1}} = \left( {1 - {\upalpha }} \right) \cdot {\text{W}}^{{\text{t}}} + {\text{V}}^{{{\text{t}} + 1}}$$

$${\text{V}}_{{\text{b}}}^{{{\text{t}} + 1}} = {\upmu } \cdot {\text{V}}_{{\text{b}}}^{{\text{t}}} - {\upeta } \cdot \nabla_{{{\text{b}}^{{\text{t}}} }} {\text{C}}$$

$${\text{b}}^{{{\text{t}} + 1}} = {\text{b}}^{{\text{t}}} + {\text{V}}_{{\text{b}}}^{{{\text{t}} + 1}}$$

where t is a discrete time-step, W are the weights, 1 − α is a regularization constant, ${\upmu }$ is the momentum constant and ${\upeta }$ is the learning rate constant. $\nabla_{{\text{W}}} {\text{C}}_{{{\text{first}}}}$ is the first computed gradient. $V$ was initialized as: $V_{0} = - {\upeta } \cdot \nabla_{W} C_{first}$.

### Figure 3d optimized parameters

**Table:** 

Momentum strategy—1 hidden layers					
Examples/digit	η	μ	α	Amp_1_	Epoch
15	0.0004	0.95	0.005	0.1	300
30	0.0054	0.978	0.0003	0.000095	300
60	0.0000089	0.9999	0.00017	0.0000975	300

**Table:** 

Momentum strategy—1 hidden layers			
Examples/digit	Epoch	Success rate	Std
15	300	0.802	± 0.0142
30	300	0.8533	± 0.0138
60	300	0.8869	± 0.0047

**Table:** 

Momentum strategy with input crosses—1 hidden layers					
Examples/digit	η	μ	α	Amp_1_	Epoch
15	0.0079	0.63	0.00018	0.07	200
30	0.008	0.773	0.00047	0.05	200
60	0.00047	0.961	0.00028	0.1	200
90	0.0003	0.99555	0.0001	0.09	200

**Table:** 

Momentum strategy with input crosses- 1 hidden layer			
Examples/digit	Epoch	Success rate	Std
15	200	0.8413	± 0.0082
30	200	0.8894	± 0.01
60	200	0.921	± 0.0025
90	200	0.9332	± 0.0032

Note that in Fig. 3d for 50,000 examples and without input crosses we obtained a test error *ε* = 0.018, which is consistent with a power law^34^. The parameters used in this optimization are $\eta = 0.03, \mu = 0.9998, \alpha = 0.0079, Amp_{1} = 0.0001, epoch = 300.$ Here we used minibatch = 200, since the optimization over the parameters with high precision was complex.

### Architecture and initial weights for FFTN, Fig. 2b

The feedforward neural network comprised of 10 identifiers, each consisted of 784 inputs that were divided into groups of 16 consecutive pixels along the rows, one hidden layer consisted of 49 units and one output unit. Each unit in the hidden and the output layers had an additional input from a bias unit. We denote by W^1^ and W^2^ the weights from the input layer to the hidden layer, and from the hidden layer to the output layer, respectively. The initial conditions of all weights were randomly chosen from a Gaussian distribution with a zero average and Std equals 1. All weights to each hidden unit were normalized^19^ such that they had a zero average and Std equals 1.

### Input

The first hidden layer was not fully connected, therefore the input ${\text{X}}$ for each hidden unit was calculated as:

$${\text{X}}_{j} = [X_{{\left( {k - 1} \right)*p + 1}} \ldots X_{k*p} ]$$

where ${\text{X}}_{j}$ represent the different input groups of jth hidden unit.

k = 1,2,.0.16 and p is a number running from 1 to $\frac{{\text{size of input }}}{16}$ .

The average test error for 50,000 examples and 50 epochs was *ε* = 0.047 and the Std was 0.023.

The test error with committee of 6 trained networks was *ε* = 0.0339 and the Std was 0.025.

The parameters were: ${\eta }$ = 0.023, ${\upmu }$ = 0.998, ${\upalpha }$ = 0.0000002, $Amp_{1}$ = 0.1

It was noted that for the case where inputs were projected randomly to each hidden unit, very similar test errors (with less than 1% decrease) were obtained but with increased Std between samples. Similar results were also obtained for a similar architecture with 56 hidden units, where each one is connected to 14 inputs instead of 16.

### Architecture and initial weights, Fig. 3e

The network comprised of 10 identifiers that each contained 784 input units with additional 10,000 input-crosses for each hidden unit (see Input), and one hidden layer consisting of 7 units each. The input and the hidden layers were fully connected, except the input-crosses. We denote by W^1^ and W^2^ the weights from the input layer to the hidden layer, and from the hidden layer the output layer, respectively. The initial conditions of W^1^ were randomly chosen from a Gaussian distribution with a zero average and Std equals 1. All weights to each hidden unit were normalized^19^ such that they had a zero average and Std equals 1.

Note that W^2^ weights were set to 1, see Fig. 3e.

### Forward and back propagation

The output of a unit, j, in the first hidden layer for the mth example, for instance, $a_{j,m}^{1}$, was calculated as:

$${\text{z}}_{{{\text{j}},{\text{m}}}}^{1} = \mathop \sum \limits_{{\text{j}}} \left( {{\text{W}}_{{{\text{ij}}}}^{1} \cdot {\text{X}}_{{\text{i}}} } \right)$$

$$a_{{{\text{j}},{\text{m}}}}^{1} = \left\{ {\begin{array}{*{20}c} {1\quad if {\text{z}}_{{{\text{j}},{\text{m}}}}^{1} > 0} \\ {0\quad otherwise } \\ \end{array} } \right.$$

where ${\text{W}}_{{{\text{ij}}}}^{1}$ is the weight from the ith input unit to the jth hidden unit, ${\text{X}}_{{\text{i}}}$ is the ith input and ${\text{b}}_{{\text{j}}}^{1}$ is the bias induced on the jth unit in the first hidden layer. ${\text{z}}_{{{\text{j}},{\text{m}}}}^{1}$ represents the field propagating from the input layer.

The weights were updated according to the following:

$${\text{W}}^{{{\text{t}} + 1}} = \left( {1 - {\upalpha }} \right){\text{W}}^{{\text{t}}} + {{ \eta }} \cdot \left( {{\text{y }} - {\text{ a}}} \right) \cdot {\mathbf{X}}$$

where t is a discrete time-step, W are the weights, 1 − α is a regularization constant, ${\upeta }$ is a constant learning rate, $y$ stands for the desired labels, and ${\text{a}}$ stands for the current output unit.

Note that the update was realized on the perceptron with a wrong output and a minimal absolute local field, and only when the number of perceptrons with the correct output was less than 5.

The average test error for 50,000 examples and 50 epochs was *ε* = 0.029 and the Std was 0.019. The parameters were: ${\eta }$ = 0.05, ${\upalpha }$ = 0.0002.

### Test accuracy

The network test accuracy was calculated based on the MNIST dataset for testing, containing 10,000 input examples. The test examples were modified in the same way as the examples in the training dataset. Reported averaged test errors and their Std are based on at least 10 samples with different initial conditions.

### Optimization

For a given architecture and number of epochs, the optimization procedure first evaluated the test error over a rough grid of the adjustable parameters, followed by fine-tuning grids with higher resolutions. In cases where a complete optimization over a grid was impossible, we optimized sequentially each parameter over its 1D grid. Nevertheless, we confirmed that a few different sequential orders of the optimized parameters resulted in the same optimized test accuracy and set of parameters.

The optimization was performed independently for each examined dataset size, number of examples and number of epochs. The hyperparameters were optimized using several validation sets. Results for the committee systems were based on the optimized selected parameters for a single system. The optimized parameters were summarized in the presented tables.

We note that cross validation was confirmed using several validation databases consisting each of 10,000 random examples with the same statistics for each label as in the test set. Averaged results had the same Std as reported test errors. Similar results were obtained using a test set with different initial conditions. In addition, preliminary results also indicate that databases consisting of random selected examples, also result in similar test errors.

### Committee

The test error was further minimized using a soft committee decision based on several replicas, *Nc*, of the network, which were trained on the same set of examples but with different initial weights. The result label, j, for the test accuracy is given by:

$$\mathop {{\text{max}}}\limits_{j} { }\left( {\mathop \sum \limits_{{{\text{s}} = 1}}^{{{\text{Nc}}}} {\text{a}}_{{{\text{j}},{\text{s}}}}^{{\text{L}}} } \right)$$

where ${\text{a}}_{{{\text{j}},{\text{s}}}}^{{\text{L}}}$ stands for the value of the output label j in output layer L and in replica s (j = 0, 1, …0.9).

### Experimental methods

The In-Vitro experimental methods are similar to those of our previous studies^43,45^, and only the modifications are presented.

### Animal use

All procedures were in accordance with the National Institutes of Health Guide for the Care and Use of Laboratory Animals and the Bar-Ilan University Guidelines for the Use and Care of Laboratory Animals in Research and were approved and supervised by the Bar-Ilan University Animal Care and Use Committee.

### Stimulations—MEA

Extracellular stimulations were applied with an amplitude of [− 900 − 200] mV and a duration of [0.2 2] ms.

### Neuronal response latency

The neuronal response latency is defined as the time-lag between a stimulation pulse onset and its corresponding evoked spike measured by crossing a threshold of − 20 mV.

### Statistical analysis

Reported results are based on 8 experiments, using different examined neuronal cultures. Presented results demonstrate an example of a decrease of 100 mV in the stimulation threshold amplitude after training. This decrease was observed in all 8 experiments. We detected stable neurons with appropriate features of stimulating electrodes (e.g. same spike waveforms with different maximal firing frequencies and neuronal responses latencies) in 8 out of 15 examined cultures.

## Data Availability

Source data are provided with this paper. All other data that support the plots within this paper and other findings of this study are available from the corresponding author upon reasonable request. A prototype simulation code in matlab for the FFTN is provided with this paper in GitHub.
